# Transgenerational exposure of North Atlantic bivalves to ocean acidification renders offspring more vulnerable to low pH and additional stressors

**DOI:** 10.1038/s41598-017-11442-3

**Published:** 2017-09-12

**Authors:** Andrew W. Griffith, Christopher J. Gobler

**Affiliations:** School of Marine and Atmospheric Sciences, Stony Brook University, Southampton, NY 11968 USA

## Abstract

While early life-stage marine bivalves are vulnerable to ocean acidification, effects over successive generations are poorly characterized. The objective of this work was to assess the transgenerational effects of ocean acidification on two species of North Atlantic bivalve shellfish, *Mercenaria mercenaria* and *Argopecten irradians*. Adults of both species were subjected to high and low *p*CO_2_ conditions during gametogenesis. Resultant larvae were exposed to low and ambient pH conditions in addition to multiple, additional stressors including thermal stress, food-limitation, and exposure to a harmful alga. There were no indications of transgenerational acclimation to ocean acidification during experiments. Offspring of elevated *p*CO_2_-treatment adults were significantly more vulnerable to acidification as well as the additional stressors. Our results suggest that clams and scallops are unlikely to acclimate to ocean acidification over short time scales and that as coastal oceans continue to acidify, negative effects on these populations may become compounded and more severe.

## Introduction

Changes in climate are occurring at rates that are unprecedented within the past ~300 My of Earth’s geological record^1–3^. The pH within the ocean surface has decreased ~0.1 pH units since the industrial revolution and is predicted to decrease an additional 0.2–0.3 units by the end of the century if carbon emission continue unabated^4^. Similarly, during the past century, mean surface ocean temperatures have risen ~1 °C and are projected to increase an additional 2–4 °C by 2100^5, 6^. Identifying how organisms respond to rapid climate perturbations over successive generations is required to understand the fate of marine organisms in climate-altered environments^7, 8^. Such investigations may reveal mechanisms by which organisms could employ to acclimate and adapt to climate change stressors^7, 9, 10^. While the body of literature regarding the effects of acidification on calcifying organisms is now extensive^6, 11^, the effects over successive generations remain unclear^8, 10^.

Transgenerational plasticity (e.g. transgenerational acclimation) refers to non-genetic inheritances passed from adults to offspring as a result of exposure to a particular perturbation, often during gametogenesis^10, 12^, and can influence how offspring respond to similar perturbations^9, 13^. Mechanisms of transgenerational plasticity include the provisioning of maternal nutrients^14, 15^ and/or epigenetic controls on gene expression^16–18^. As a result of transgenerational plasticity, offspring may exhibit decreased sensitivity to stressors, especially during early-life stages^9^. Such plastic responses may sustain populations in the presence of persistent stress until beneficial mutations and/or environmental pressures select for individuals that are better adapted to such environments^19^. However, parental exposure to environmental stress does not always confer resistance in subsequent generations as carryover effects can render offspring more sensitive to stressors^10, 20^. In addition, if environmental conditions improve (i.e. seasonal changes in temperature, dissolved oxygen, pH etc.) during early life-stages, next-generation phenotypes may be unsuitably adapted to stress-free environments^13, 21^. While transgenerational plasticity has been observed in several marine invertebrates^8^, this phenomenon is likely to be highly species-specific and will depend largely upon the magnitude and duration of adverse conditions.

Recently, the effects of exposure to elevated *p*CO_2_ during reproductive conditioning on next-generation *Saccostrea glomerata* (Sydney rock oyster) were investigated^7^ and the carbonate chemistry in which parents were exposed to during gametogenesis was found to influence larval fitness. Larvae originating from adults reproductively conditioned within acidified conditions (pH = 7.7; *p*CO_2_ = 856 μatm), regardless of larval carbonate chemistry, exhibited more rapid growth and development than larvae resulting from parents conditioning in ambient pH regimes^7^, a potential indication of acclimation. However, pH and *p*CO_2_ levels utilized in this study were mild in comparison to worst-case climate change projections^2^ and to conditions that are already present in some coastal ecosystems^22–24^. More recently, Thomsen *et al*. ^25^ observed that mussels originating from environments naturally enriched in CO_2_ displayed greater adaptive potential and increased fitness when grown in acidified environments than larvae originating from non CO_2_-enriched environments. While there is a growing body of literature exploring the transgenerational effects of ocean acidification on marine organisms, impacts on bivalves including those inhabiting the North Atlantic have been poorly studied.

Organisms in future oceans will likely be exposed to multiple, co-occurring stressors including thermal stress, acidification, and lower dissolved oxygen (DO) levels^26, 27^. In coastal areas, some of these stressors are already present as low oxygen conditions often occur in unison with low pH due to accelerated rates of organic matter decomposition and microbial respiration^23, 28, 29^. Within these systems, pH levels are already at or below levels that are predicted to occur in open-ocean systems by the end of this century^22, 23^. Additionally, increases in ocean temperatures in temperate-latitude regions have made these areas more favorable for several species of harmful or toxic algae^30, 31^. Temperature induced stratification of the water column may also render surface layers nutrient limited, a condition that may reduce productivity within the sea surface and decrease the food available for organisms feeding at or near the base of marine food webs^32, 33^. Therefore, the occurrence of multiple, co-occurring stressors in coastal systems is common^23, 24, 27^ and may be more common in the future. Identifying potential synergistic, additive, and antagonistic effects of multiple stressors on marine life will provide more accurate predictions regarding their fate in future oceans^27^.

The purpose of this study was to identify transgenerational effects of ocean acidification within two species of North Atlantic bivalve molluscs, *Mercenaria mercenaria* (=hard clam; northern quahog) and *Argopecten irradians* (bay scallop). Adult clams and scallops were collected from native populations from Long Island (NY, USA) and exposed to low and ambient pH throughout gametogenesis. Larvae from each parental cohort were exposed to ambient and low pH environments as well as additional stressors including increased temperature, food-limitation, and exposure to a harmful alga (*Cochlodinium polykrikoides*) to discern the effects of parental and early larval carbonate chemistry on the development of offspring.

## Results

### Hard clams – *Mercenaria mercenaria*

**Figure 1:**
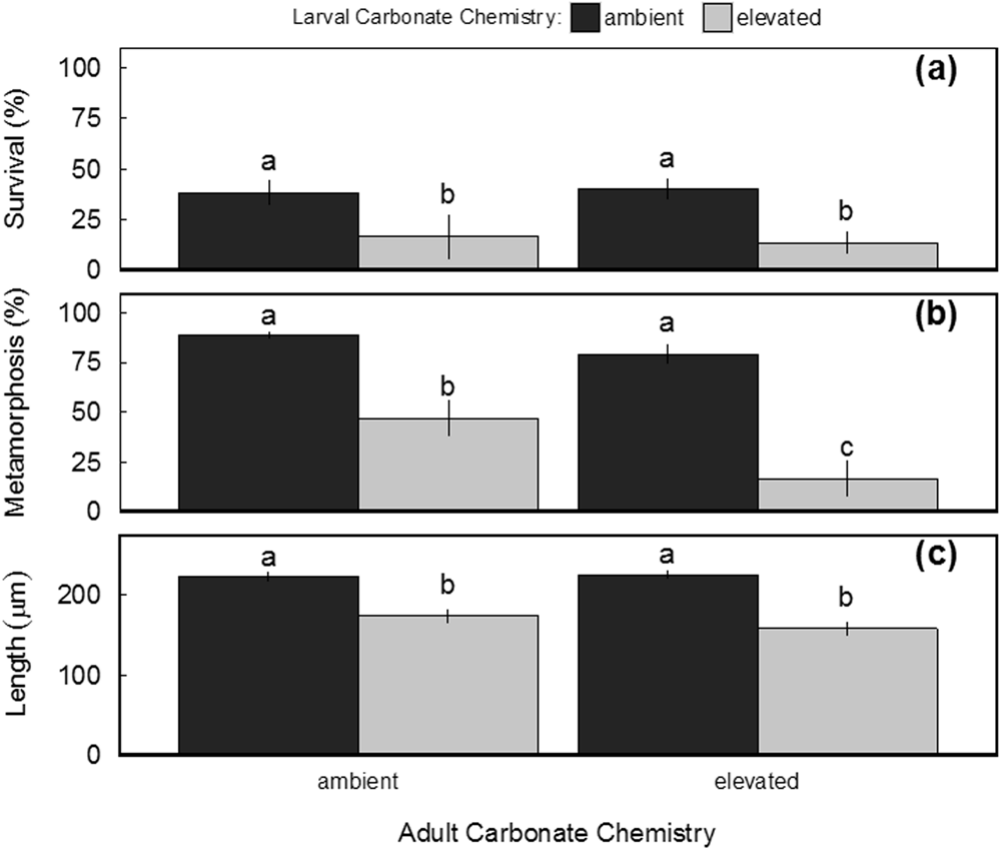
Final survival (**a**), development (**b**), and size (**c**) of larval hard clams after 19-days development in acidification trials (*n* = 4, error bars denote ± S.D.; letters denote significant groupings; *p* < 0.05; two-way ANOVA).

The level of seawater acidification that hard clam larvae were reared in had a significant effect on their survival (F_1,11_ = 38.893, *p* = 6.38 × 10^−5^; two-way ANOVA; Fig. 1a) while the carbonate chemistry of seawater in which their parents underwent reproductive conditioning did not (F_1,11_ = 0.062, *p* = 0.808; two-way ANOVA). Survival of clam larvae originating from ambient CO_2_-treatment adults and reared in ambient pH was nearly two-fold greater than clams originating from the same parents but exposed to low pH (*p* = 0.013; Tukey HSD; Fig. 1a). Survival of larvae from elevated CO_2_-treatment adults exhibited similar patterns, with larvae reared under low pH displaying significant reductions in survival relative to ambient pH counterparts (*p* = 1.99 × 10^−3^; Tukey HSD; Fig. 1a). Survival rates of both larval cohorts reared within low pH environments were not statistically different (*p* = 0.945; Tukey HSD).

Carbonate chemistry during both reproductive conditioning and early-larval development had significant independent (F_1,11_ = 43.109, *p* = 4.01 × 10^−5^ and F_1,11_ = 35.717, *p* = 6.45 × 10^−5^, respectively; two-way ANOVA) and interactive effects (F_1,11_ = 9.93 × 10^−9^, *p* = 0.011; two-way ANOVA) acting to delay clam development (Fig. 1b). Among larvae from ambient CO_2_-treatment adults, those reared within low pH exhibited significant delays in metamorphosis after 19 days (*p* = 2.90 × 10^−5^; Tukey HSD). Development rates of larvae from elevated CO_2_-treatment adults and exposed to low pH during early-life were further decreased and significantly lower than rates of both cohorts of larvae originating from ambient CO_2_-treatment adults (*p* = 1.00 × 10^−8^ and *p* = 4.89 × 10^−4^; Tukey HSD) and to larvae from the same parents but reared in ambient pH (*p* = 2.00 × 10^−8^; Tukey HSD; Fig. 1b).

Beyond impacts on survival and development, the carbonate chemistry in which larvae were reared had significant independent (F_1,11_ = 200.796, *p* = 2.07 × 10^−8^; two-way ANOVA) and interactive (F_1,11_ = 5.399, *p* = 0.043; two-way ANOVA) effects with adult carbonate chemistry upon the final size of larvae. Cohorts of larvae reared under low pH originating from ambient CO_2_- or elevated CO_2_-treatment adults were significantly (*p* = 3.63 × 10^−5^ and *p* = 6.00 × 10^−8^, respectively; Tukey HSD) smaller than larvae from the same parents but reared under ambient pH (Fig. 1c). There was no effect of pH on the size of eggs released by adult female hard clams (Welch’s t-test; data not shown).

**Figure 2:**
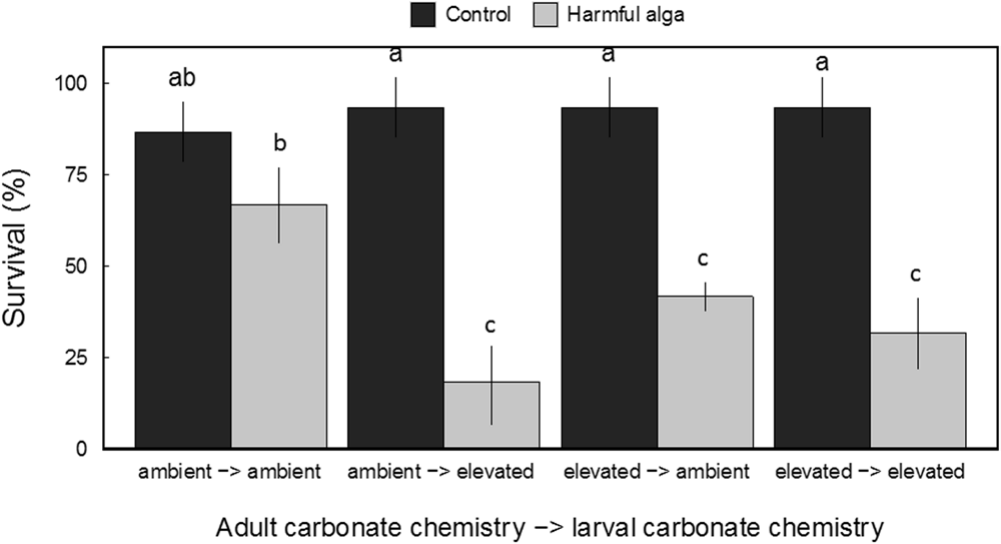
Survival of hard clam larvae (from each experimental cohort) after 9-days exposure to *Cochlodinium polykrikoides* (*n* = 6, error bars denote ± standard deviation; letters denote significant groupings; *p* < 0.05; three-way binomial GLM).

Exposure to a harmful alga (*C. polykrikoides*) significantly reduced the survival of larval clams (Z = −2.517, *p* = 0.011; three-way GLM; Fig. 2). In addition, adult and larval carbonate chemistry interacted significantly with *C*. *polykrikoides* exposure (Z = −2.411, *p* = 0.015 and Z = −3.819, *p* = 0.025 respectively; three-way GLM) to further reduce overall survival. Specifically, larvae exposed to low pH during early development (48 h) only or arising from elevated CO_2_-treatment adults displayed greater sensitivity to *C. polykrikoides* than larvae from ambient CO_2_-treatment adults reared in ambient pH (Fig. 2). Among larvae exposed to C. *polykrikoides*, the groups exhibiting the greatest survival were those from ambient CO_2_-treatment adults reared at ambient pH and were significantly (all *p* < 0.05; Tukey HSD) greater than the survival of all remaining larval cohorts (Fig. 2). Survival rates of larvae from elevated CO_2_-treatment adults subjected to ambient pH during early development yielded less than half the survival rate of larvae from ambient CO_2_-treatment adults subjected to ambient pH (Fig. 2). The lowest rates of survival were exhibited by larvae originating from ambient CO_2_-treatment adults subjected to low pH during early development and exposed to *C. polykrikoides* (after 9 days of exposure; see Fig. 2).

**Figure 3:**
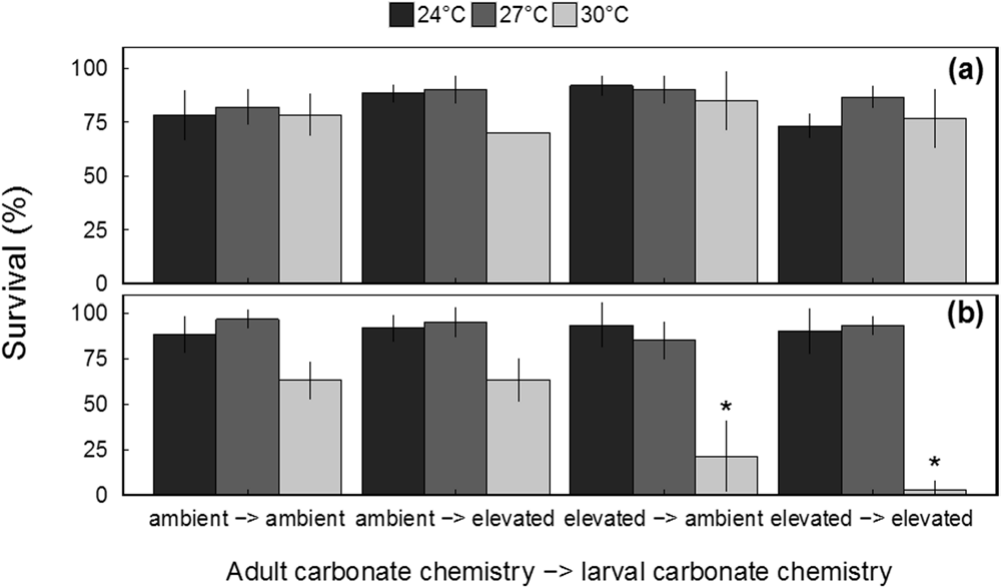
Survival of larval hard clams exposed to increased temperature with (**a**) and without (**b**) the addition of *Isochrysis* spp. (*n* = 6, error bars denote ± standard deviation; * indicates significant differences from all remaining treatments; *p* < 0.05; three-way GLM).

During trials with fed larvae exposed to increased temperature, marginal (Z = 2.018, *p* = 0.043; three-way GLM) effects of larval carbonate chemistry on survival were observed whereby larvae exposed to low pH during early development (48 h) yielded slight, but significantly, elevated rates of survival. In addition, minimal interactive effects between temperature and both early-larval (Z = −1.961, *p* = 0.49; three-way GLM) and adult carbonate chemistry (Z = −2.095, *p* = 0.03; three-way GLM; Fig. 3a) treatments were observed with survival, again, being slightly greater among larvae exposed to increased temperature (i.e. 27 and 30 °C) resulting from or reared at low pH.

Within food-limited treatments, temperature (Z = −3.438, *p* = 5.86 × 10^−4^; three-way GLM) and adult carbonate chemistry (Z = 2.774, *p* = 5.53 × 10^−3^; three-way GLM) had significant independent and interactive effects (Z = −3.044, *p* = 2.33 × 10^−3^; three-way GLM; Fig. 3b) on overall larval survival. Survival of clam larvae from elevated CO_2_-treatment adults exposed to ambient or low pH during early development and starved at 31 °C for 1 week was significantly (all *p* < 0.01; Tukey HSD) reduced relative to the survival within the remaining treatments at each temperature, of which all were above 60% (Fig. 3b). These effects were absent when larvae were fed an optimal diet of *Isochrysis* spp. (3 × 10^4^ cells mL^−1^ d^−1^; Fig. 3a).

### Bay scallops – *Argopecten irradians*

**Figure 4:**
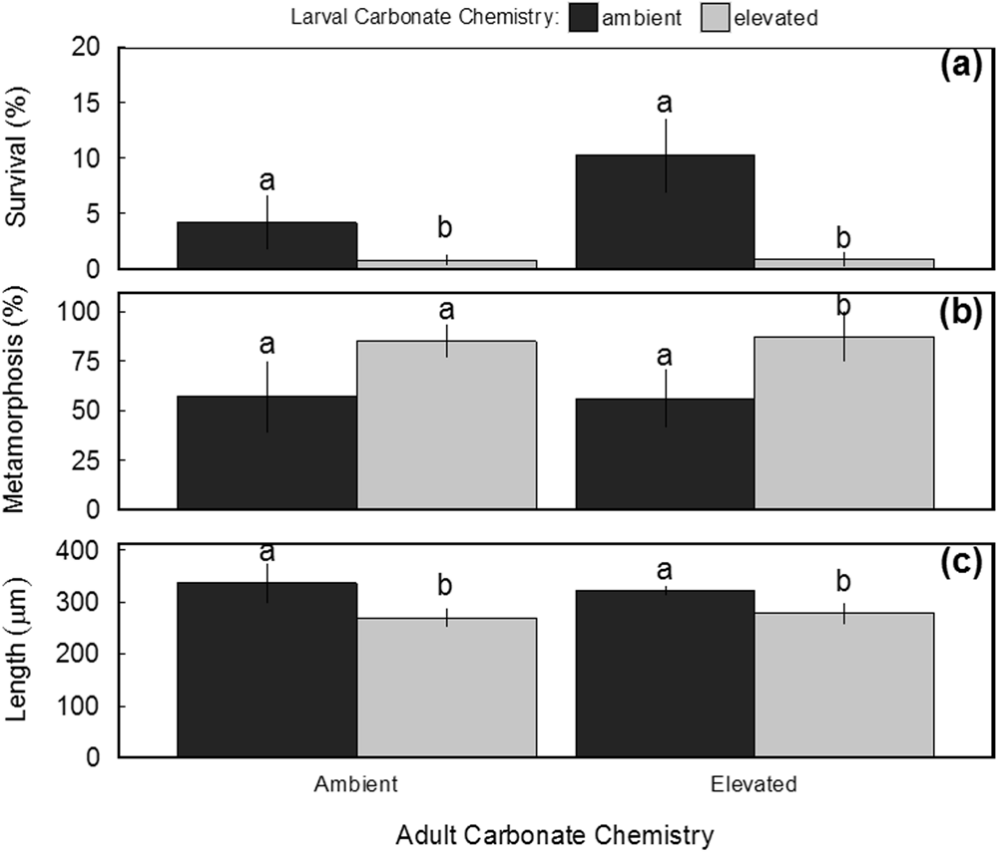
Final survival (**a**), development (**b**), and size (**c**) of larval bay scallops after 19-days development in acidification trials (*n* = *4*, error bars denote ± standard deviation; letters denote significant groupings; *p* < 0.05; two-way ANOVA).

In a manner similar to clams, there were significant effects of adult and larval carbonate chemistry (F_1,12_ = 36.009, *p* = 6.21 × 10^−5^ and F_1,12 = _8.368, *p* = 0.0135; two-way ANOVA) as well as significant (F_1,12_ = 7.989, *p* = 0.0153) interactive effects on the survival of larval scallops (Fig. 4a). Larvae from ambient and elevated CO_2_-treatment adults exposed to normal carbonate chemistry conditions displayed survival rates (post-metamorphosis) that were significantly elevated relative to larvae reared within low pH treatments (*p* = 0.05, and *p* = 0.006; respectively; Tukey HSD; Fig. 4a). With regards to development, the effects of larval carbonate chemistry were also significant (F_1,12_ = 18.381, *p* = 1.06 × 10^−3^; two-way ANOVA) with larvae reared under low pH regimes and originating from elevated CO_2_-treatment adults exhibiting significantly greater rates of metamorphosis than both cohorts of larvae reared under ambient pH (*p* = 0.039 and *p* = 0.032 respectively; Tukey HSD; Fig. 4b). By day 19, larvae from ambient CO_2_-treatment adults exposed to low pH displayed slightly accelerated rates of development, but were not significantly (*p* = 0.10) different than rates displayed by larvae from the same parents exposed to ambient pH (Fig. 4b). Any slight, yet significant, increases in metamorphosis may be likely a result of lower survivorship among larval scallops. Carbonate chemistry during larval phases had significant (F_1,11_ = 21.352, *p* = 7.39 × 10^−4^; two-way ANOVA) impacts on the final size of scallop larvae (Fig. 4c), with cohorts of larvae exposed to low pH during early development exhibiting smaller overall size (*p* = 7.53 × 10^−4^; Tukey HSD; Fig. 4c). There was no effect of carbonate chemistry on the diameter of scallop eggs (Welch’s t-test; data not shown).

**Figure 5:**
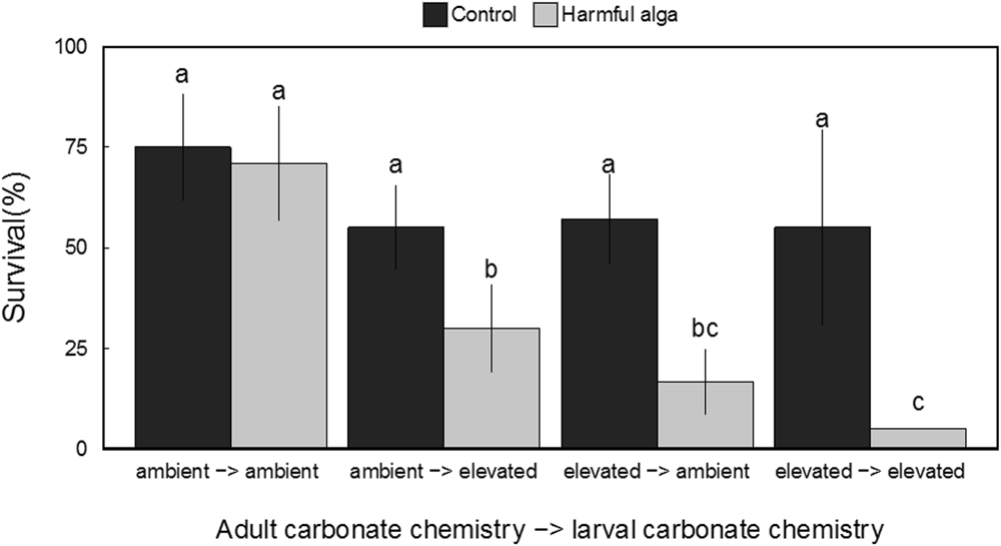
Survival of scallop larvae (from each experimental cohort) after 9-days exposure to *Cochlodinium polykrikoides* (*n* = 6, error bars denote ± standard deviation; letters denote significant groupings; *p* < 0.05; three-way GLM).

Exposure of scallops to acidified conditions during gametogenesis and early-larval development (48 h) significantly increased their vulnerability to additional stressors. During experiments with *C. polykrikoides*, adult and larval carbonate chemistry had significant independent (Z = −2.174, *p* = 0.029 and Z = −2.174, *p* = 0.029 respectively; three-way GLM) effects on scallop survival. In addition, interactive effects between adult carbonate chemistry and *C. polykrikoides* on larval survival were observed (Z = −2.757, *p* = 5.83 × 10^−3^; three-way GLM; Fig. 5). Survival rates among both larval cohorts originating from elevated CO_2_-treatment adults or those exposed to low pH only during early development, when exposed to *C. polykrikoides* (~3 days), were significantly (*p* = 1.0 × 10^−3^; Tukey HSD) lower than rates observed among larvae from ambient CO_2_-treatment adults reared at ambient pH or larvae not exposed to the harmful alga (Fig. 5).

**Figure 6:**
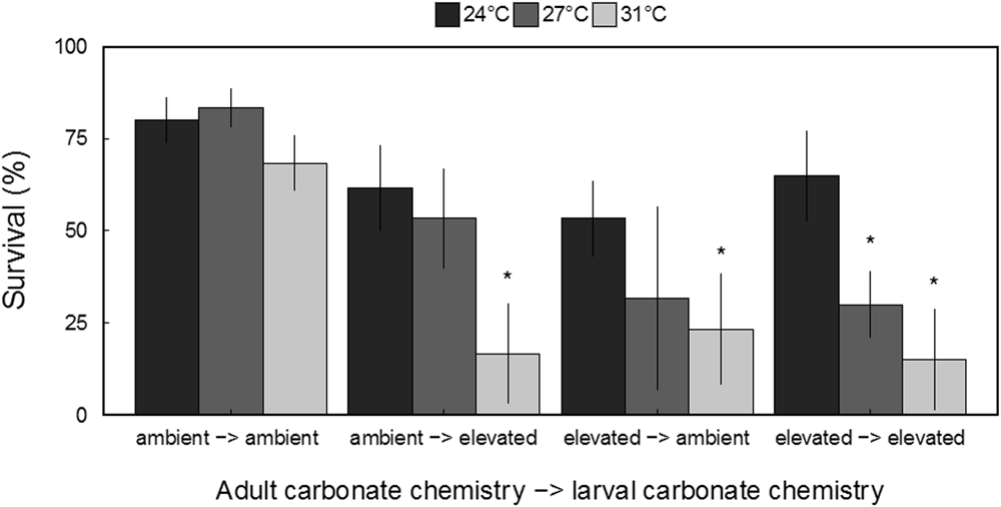
Survival of larval scallops exposed to increased temperature (*n* = 6, error bars denote ± standard deviation; * denote significant differences relative to control (e.g. 24 °C) within each experimental cohort; *p* < 0.05; three-way ANOVA).

Within temperature and food-limitation trials, temperature (F_2,60_ = 32.257, *p* = 3.08 × 10^−10^; three-way ANOVA), adult carbonate chemistry (F_1,60_ = 41.370, *p* = 2.31 × 10^−8^; three-way ANOVA), and larval carbonate chemistry (F_1,60_ = 21.351, *p* = 2.08 × 10^−5^; three-way ANOVA) were found to have significant effects on larval survival (Fig. 6). In addition, significant interactive effects between adult and larval carbonate chemistry (F_1,60 = _23.037, *p* = 1.090 × 10^−5^; three-way ANOVA), adult carbonate chemistry and temperature (F_2,60 = _4.443, *p* = 0.0158; three-way ANOVA), and larval carbonate chemistry and temperature (F_2,60_ = 5.867, *p* = 4.71 × 10^−3^; three-way ANOVA) were observed. When exposed to thermal stress (31°C) and concurrent food-limitation, survival of scallop larvae from elevated CO_2_-treatment adults exposed to ambient and low pH as larvae or exposed to low pH during early development only were significantly (all *p* < 0.05; Tukey HSD) lower than larvae originating from ambient CO_2_-treatment adults reared at ambient pH (i.e. control treatment; Fig. 6). At moderately increased temperature (e.g. 27°C) the only treatment to display significant (*p* = 1.0 × 10^−3^; Tukey HSD) reductions in survival relative to larvae within control treatments was from elevated CO_2_-treatment adults reared at low pH (Fig. 6).

## Discussion

Bivalve larvae experiencing ocean acidification grow more slowly^34, 35^, display higher rates of abnormality^36, 37^, and experience lower survivorship compared to larvae grown under optimal pH conditions^28, 34, 38^. These observations, however, emanate from experiments in which larvae were spawned from adults that were reproductively conditioned in ideal environments (i.e. ambient pH), a scenario that may not be representative of future climate change or estuaries experiencing coastal acidification today. Such single-generation investigations may not accurately represent the long-term consequences of climate change, especially when multiple stressors are present.

During this study, transgenerational acclimation was not observed. Bivalve larvae originating from adults undergoing reproductive conditioning within low pH environments produced larvae that were as or more sensitive to low pH than larvae originating from ambient pH-treatment adults, indicating beneficial phenotypic changes are unlikely to occur within these organisms over single generations. Given the accelerating pace of climate change^3, 5, 6^ as well as the rapid seasonal onset of coastal acidification^22, 23, 29^, the window of opportunity for bivalves to acclimate or adapt may be limited. Selective pressures may become progressively stronger on these bivalves in subsequent generations, as larvae originating from elevated CO_2_-treatment adults displayed increased sensitivities to other environmental stressors including thermal stress and harmful algae. Further, it is possible that extended exposure of adult bivalves to low pH beyond gametogenesis would have an even more severe effect on their performance and offspring.

In the absence of beneficial epigenetic inheritances or rapid acclimation, organisms may rely upon evolutionary (e.g. genetic) responses to cope with climate changes. However, these changes require an extended period of time to begin to affect responses at population levels^10^. Species with shorter generational (i.e. weeks to months) times may have a greater ability for adaption, whereas longer-lived organisms (i.e. molluscs) with longer generational (i.e. years) times may have a lowered capacity to do so^8, 10^. The bivalves studied here have biogeographic ranges from Canada to the Gulf of Mexico^39, 40^. Within the southern extent of this distribution, the solubility of CO_2_ is relatively low and the buffering capacity of water is high (i.e. LA, TX, FL; ref. 41), conditions that may lead to a slower rate of acidification and thus more time to acclimate/adapt to changes in carbonate chemistry. In contrast, some eutrophic estuaries within the Northeast US already experience acidification^23^, have warmed more rapidly^31^, are predicted to warm faster in the future^42^, and be more susceptible to atmospherically driven ocean acidification^41^. Hence, shellfish within these temperate-locales are likely more vulnerable to climate change stressors given current conditions and accelerating rates of change^43^. However, patterns of adaptation are likely to be species-specific and will be, in part, dependent upon the persistence and magnitude of climate changes in addition to the presence of additional stressors.

Species-specific responses of bivalves to stressors reported here are consistent with prior studies. Among the shellfish transgenerationally exposed to acidification and subjected to moderate thermal stress (e.g. 27°C), only bay scallop larvae displayed significantly lowered survival (see Fig. 6), suggesting an increased sensitivity to thermal stress. These observations are consistent with other studies of environmental stressors and shellfish where bay scallops have been found to be more sensitive to acidification^34, 44^, low dissolved oxygen^28, 45^, and exposure to harmful algae^46, 47^. In addition, low survivorship is also a common phenomenon among cultured bay scallops (J. Dunne, East Hampton Town Shellfish Hatchery, Montauk, NY, USA, and G.J. Rivara, Cornell University Cooperative Extension, Southold, NY, USA, personal communication). Collectively, these findings suggest the impacts of climate change may be more severe on bay scallops than other populations of North Atlantic bivalves.

### Transgenerational effects of acidification on shellfish

Recent transgenerational investigations with bivalve shellfish suggest some species can acclimate to ocean acidification^7, 25, 48^. Experiments with selectively and non-selectively bred Sydney rock oysters revealed parental carryover effects in next-generation larvae^7^. Larvae from oysters undergoing reproductive conditioning within elevated *p*CO_2_ environments grew faster and larger than larvae originating from ambient *p*CO_2_ parents^7^, a possible indication of transgenerational acclimation. In addition, *Mytilus edulis* larvae originating from high CO_2_ environments have displayed increased tolerance to acidified environments^25^, and have, in some cases, been observed to no longer precipitate aragonite, but rather calcite, a less-soluble form of CaCO_3_, a change that may represent long term adaptation^48^.

In contrast to these prior studies, results presented here demonstrate that larvae spawned from *M. mercenaria* and *A. irradians* reproductively conditioned in elevated *p*CO_2_ environments are as or more sensitive to acidification than individuals originating from adults conditioned under ambient *p*CO_2_. Offspring from both clams and scallops subjected to acidification (e.g. pH_T_ = 7.4) during gametogenesis produced larvae that displayed rates of survival similar to those originating from adults conditioned at ambient pH levels (e.g. 7.9). There are several factors that could account for differences between the results obtained here and prior studies^7, 48^ including species and/or strain differences, as well as the levels of acidification used during experiments. The levels of acidification used during this study mimicked future open-ocean acidification as well as modern day coastal acidification (e.g. pH_T_ < 7.4; > 2000 μatm CO_2_; refs 22, 23, and 29) but were more extreme than levels utilized in other transgenerational studies^7, 48^. High *p*CO_2_ (800–1,000 ppm) treatments used by other groups^7, 48^ are consistent International Panel of Climate Change^2^ projections for the end of this century (*ca*. 2100) within pelagic systems but are well below levels found in some eutrophic coastal habitats today^22, 23, 29^. These conditions typically manifest during spring^22, 28^ when bivalve shellfish undergo reproductive conditioning^49^ and can persist well into summer months when the early-life stages of bivalve shellfish are present. Therefore, the high *p*CO_2_ treatments used here, while elevated compared to end-of-century open-ocean acidification^2^, are a closer approximation of conditions in current and future coastal environments and may partly explain differences in organismal responses between this study and prior studies^7, 48^.

### Mechanisms for increased sensitivity to acidification and other stressors

Recently, Sydney rock oyster larvae originating from adults undergoing gametogenesis within high *p*CO_2_ environments were found to be more sensitive to thermal stress, reduced salinity, and a limited diet than larvae from adults that reproductively conditioned under ideal *p*CO_2_ conditions^50^. Consistent with this finding, larvae spawned from adults undergoing gametogenesis within acidified environments during this study were more sensitive to acidification and other stressors including food-limitation, elevated temperature, and exposure to a harmful alga. Within elevated temperature and food-limitation trials, survival of starved larvae was significantly reduced amongst cohorts originating from parents exposed to low, but not normal, pH during gametogenesis. Similarly, hard clam larvae originating from elevated CO_2_-treatment adults and exposed to low pH during early-development were increasingly sensitive to low pH in terms of metamorphosis, outcomes potentially linked to poor maternal provisioning^14, 15^. This outcome may have been even more severe on the total population as metamorphosis could only be assessed on live individuals and among survivors full metamorphosis may have been more likely to occur.

Previous transgenerational investigations have demonstrated that non-genetic inheritances such as maternal provisioning of nutrients can have large effects on the ability of organisms to withstand environmental stress upon hatching^21, 51^. Provisioning can result in larger or more nutrient-enriched eggs, but can also come at the cost of reduced fecundity^51^. Larger eggs, however, may not necessarily reflect higher nutritional quality and may simply be composed of a greater proportion of energy-poor constituents (i.e. water; refs 52 and 53). While no evidence of differential maternal provisioning among Sydney rock oyster larvae from high and ambient *p*CO_2_ treatment-adults was observed^50^ and while the egg sizes of ambient and elevated cohorts in the current study did not differ for either species, it is plausible that the more severe levels of acidification presented here resulted in eggs that were lower in nutritional quality (e.g. lipids; ref. 52) and thus more vulnerable to stressors during the larval phase (e.g. acidification, increased temperature, food limitation, poor food).

Maintaining homeostasis under stress increases energy requirements for invertebrates^54^ and thus, adults undergoing gametogenesis under acidification may provision fewer resources within their gametes. As external food supplies become limiting, internal stores of energy become increasingly important for growth and survival of early life stage bivalves^55^. Furthermore, several studies have found that when nutrition is limited, the negative effects of acidification and other stressors can be intensified^56^. As coastal oceans warm, nutrient acquisition rates by phytoplankton will accelerate^57^ and nutrient levels may decrease. Nutrient limitation and temperature-enhanced herbivory^58^ may reduce phytoplankton biomass. Thus, organisms feeding at or near the base of marine food webs may be food-limited in future, warmer, coastal oceans^59^. Results presented here suggest that larval shellfish originating from these locales will be increasingly sensitive to co-stressors such as acidification and elevated temperatures.

Recent work has demonstrated that exposure to low pH can increase respiratory rates of bivalve larvae^35^. In many organisms a byproduct of respiratory processes are reactive oxygen species (ROS) that have the ability to wrought significant extracellular and intracellular damage^60^. As a result, many organisms have evolved antioxidant pathways that include the production of enzymes capable of neutralizing ROS, mechanisms that may become an increasingly important component of cellular self-defenses for individuals with increased respiration rates associated with exposure to low pH^35^. While the purported mode of toxicity associated with the harmful alga *C. polykrikoides* has been debated (see refs 61 and 62), the majority of evidence indicates elevated levels of ROS cause the lethal effects^61, 63, 64^. Larvae with increased respiration rates as a result of exposure to low pH during gametogenesis and/or early development may be more sensitive to additional oxidative stressors, such as those produced by *C. polykrikoides*, as cellular defense mechanisms may already be over-burdened with the additional stress (i.e. ROS production) of higher respiratory demand^35^. Such an outcome would account for the significantly elevated mortality among larvae exposed to low pH during gametogenesis and/or early development and then subjected to *C. polykrikoides*.

Future-coastal marine ecosystems are expected to host multiple, co-occurring, stressors^6^. Many acidified coastal areas also experience hypoxia^22, 23^, a co-stressor that can have additive and synergistic impacts on the development and survival of early life-stage marine bivalves^28^. In addition to chronically-low pH and DO, large natural diel variability of both parameters occur^22^ and such diurnal fluctuations of DO and pH can have a more adverse impact on the performance of larval shellfish than chronically low levels of these stressors^45^. As climate changes progress, many coastal habitats are also expected to become more favorable environments for several species of harmful algae^31^ some of which are harmful to shellfish^63, 64^. Results presented here suggest that future bivalve populations experiencing acidification during gametogenesis will be more vulnerable to other environmental stressors.

### Implications for future environments

Bivalve shellfish are ecosystem engineers and a key economic resource in coastal zones^43^. Shellfish provide essential habitat for a variety of marine organisms^65^, sequester excess nutrients from the water column^66^, and can protect coastlines from erosion and sea level rise^65^. Many harmful algal species that can be lethal to a variety of marine organisms can be effectively mitigated by filter-feeding bivalves when present at moderate densities^67^. As environmental conditions become less favorable for bivalves, the environmental benefits associated with their populations may, in turn, decline. In the field less than 1% of spawned larvae reach post-settlement stages^39^ and whole population models indicate that current ocean acidification has already caused declines in populations of the bivalves studied here^68^. Transgenerational exposure to acidification and other stressors may further limit bivalve recruitment in these locales.

Investigations that span more than a single generation better represent potential impacts of climate changes on marine organisms and may indicate how organisms will develop resistance to such changes. Transgenerational effects of climate change are likely to be species-specific and will partly depend upon the magnitude and duration of adverse conditions. While prior transgenerational investigations with bivalve shellfish suggest these organisms may be capable of acclimating to acidified environments^7, 48^, this study, that utilized environmentally realistic levels of acidification, demonstrates that transgenerational acclimation over short time-scales (i.e. single generations) is unlikely to occur in two species of North Atlantic bivalves with contrasting life-histories. Rather, as acidification continues, adults will generate offspring that are more sensitive to other stressors putting the economic and ecological benefits arising from abundant shellfish populations at risk.

## Materials and Methods

### Broodstock conditioning

This study focused on two bivalves species found across most of the east coast of North America with contrasting life histories. *A. irradians* is typically found among eelgrass beds and sandy substrates, reaches maturity at age one, spawns during summer and/or fall when seawater temperatures range from 20–24 °C, and has a lifespan of 20–26 months^69^. In contrast, *M. mercenaria* buries itself just below the sediment surface, typically reaches sexual maturity after 2–3 years, spawns once annually when water reaches optimal temperature (20–24 °C), and can live up to forty years^70^. For this study, adult bay scallops (~1 year old; ~45mm) were collected from Shinnecock Bay (NY; 40.862°N, −72.494°W) and adult hard clams (~70 mm) were obtained from Long Island Sound (Huntington, NY; 40.947°N, −73.947°W) during late winter/early spring 2015 prior to any natural significant reproductive development. These specific collection locations are mesotrophic and do not experience intense seasonal acidification^23^. Visual inspection of the gonadal tissues of multiple individuals from both cohorts of bivalves revealed no significant gonadal development prior to the commencement of experiments.

Individuals from each species were randomly assigned to low (pH_T_ ~7.4; *p*CO_2_ ~2500 μatm; Ω_aragonite_ < 1) and ambient (pH_T_ ~7.9; *p*CO_2_ = ~600 μatm; Ω_aragonite_ > 1.6) pH treatments in 70 L aquaria (n = 2 tanks treatment^−1^; 6 scallops aquaria^−1^; 10 clams aquaria^−1^) filled with filtered (1μm) seawater (see Supplementary Table S1). The chemistry within the acidified treatments used for experiments was more extreme than conditions predicted for the open ocean at the end of the century but are consistent with conditions found in eutrophic estuaries that have formerly hosted dense populations of the bivalves studied here^22, 23, 68^. Twenty liters from each tank were exchanged every other day, ensuring complete exchanges weekly. Broodstock were fed 5–7% of their dry weight daily with a mixture of live-cultured microalgae (*Isochrysis* spp., *Chaetoceros muelleri*, *C. calcitrans*, *Pavlova lutheri*, *Tetraselmis suecica* (scallops only), and *T. chuii* (scallops only); ref. 71), continuously added (~2 mL min^−1^) throughout the conditioning period using a multi-channel, peristaltic pump (Cole Parmer®). Clams and scallops were reproductively conditioned for eight and four weeks, respectively, time frames adequate for full gametic development in both species^71, 72^.

Carbonate chemistry (Supplementary Table S1) and pH within conditioning aquaria was controlled via the addition of concentrated (5%) CO_2_ gas mixed with ambient air using multi-channel gas proportioners (Cole-Palmer©). Temperature and pH_T_ measurements were made daily using a DuraFET III (Honeywell) ion-sensitive field-effect transistor-based (ISFET) solid-state pH sensor. Bi-weekly, samples were obtained for dissolved inorganic carbon (DIC) analysis using an EGM-4 Environmental Gas Analyzer® (PP systems) after acidification and separation of gas phases using a Liqui-cel® Membrane (Membrana). Saturation states for both aragonite (Ω_aragonite_) and calcite (Ω_calcite_) in addition to *p*CO_2_ were calculated from levels of total DIC, pH, pressure, temperature, salinity, phosphate, silicate, and first and second disassociation constants for estuarine waters as determined by Millero *et al*. (ref. 73) using CO_2_SYS (http://cdiac.ornl.gov/ftp/co2sys/). Certified reference material (provided by Andrew Dickson, Scripps Institution of Oceanography) was analyzed before and after analysis of experimental samples as a quality assurance measure and provided 104 ± 5% recovery.

### Bivalve spawning and larval trials involving acidification

**Figure 7:**
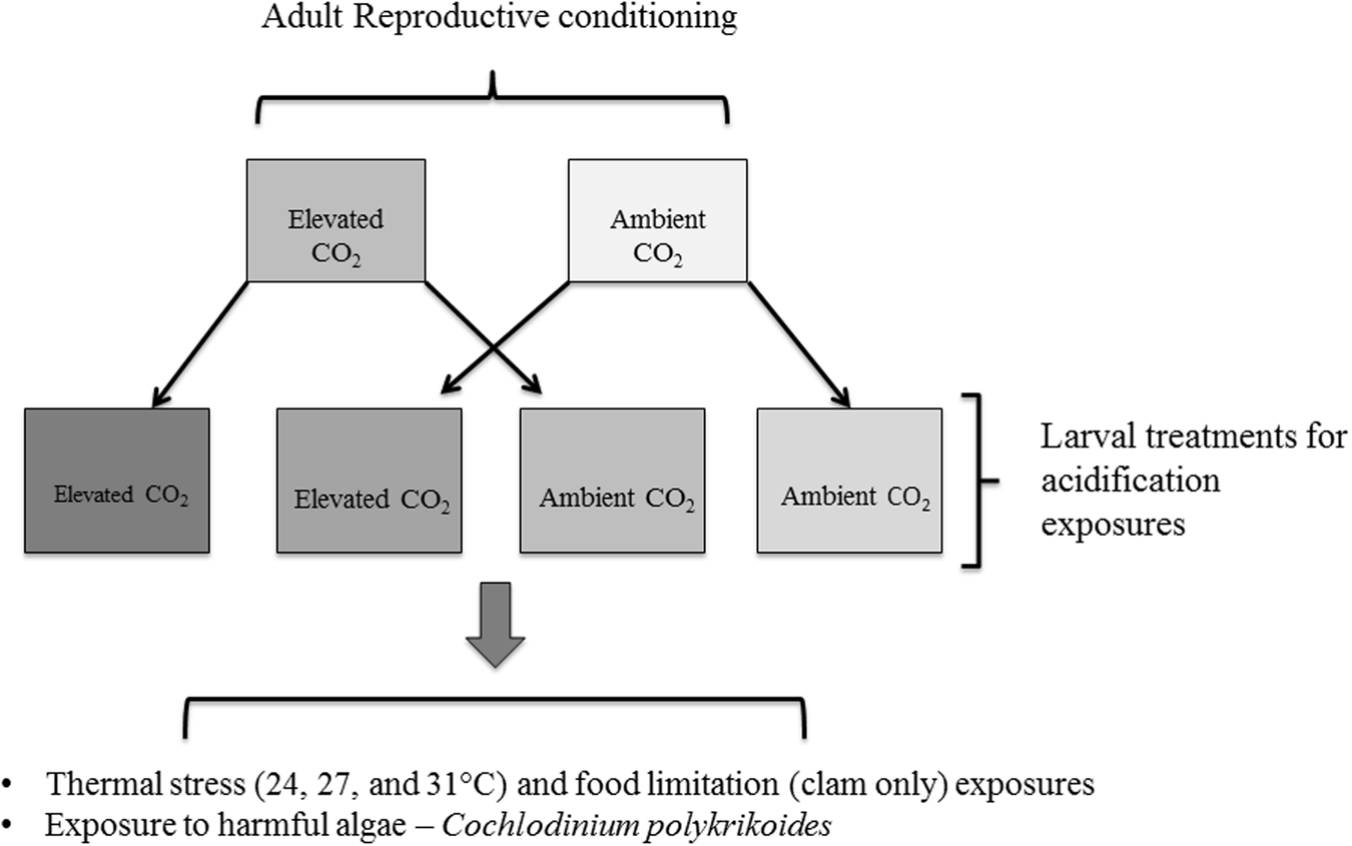
Experimental design for adult and larval experiments.

Following the conditioning period, adults were induced to spawn via thermal shock. Briefly, individuals were placed into 2-L glass dishes filled with 0.2 μm filtered-seawater and placed into temperature controlled water baths. Temperatures within the water baths were increased (~28°C) to induce the release of gametes. Clams were placed into individual spawning dishes during the release of gametes. Due to their hermaphroditic nature, bay scallops were transferred to separate spawning dishes as they switched from sperm to egg release to avoid self-fertilization. Eggs from each individual were rinsed, pooled, and re-suspended in filtered seawater (0.2 μm). Subsets of eggs collected from each female were preserved in a 3% (v/v) phosphate buffered formalin solution for quantification of egg size (detailed below). Sperm collected from each individual were pooled, carefully passed through a 20 μm mesh, and added to egg suspensions (2–3 mL sperm L^−1^ of egg suspension). Prior to fertilization, gametes were microscopically inspected for quality. Sperm were examined for motility and egg shape and size was confirmed using a light microscope. All gametes used in experiments reported here were deemed viable. After 3–4 h, viable embryos (e.g. morula-stage embryos) were added to 2-L polyethylene, experimental vessels (350 larvae L^−1^; *n* = 4 replicates treatment^−1^). Larval acidification experiments were fully factorial, including each possible parental and early-life carbonate chemistry combination (Fig. 7). Experiments were concluded once a majority of larvae had metamorphosed within a given treatment (19 days for both species).

Carbonate chemistry within experimental vessels was controlled as described above (see Supplementary Tables S2 and S3). An antibiotic solution (1% v/v final concentration; 10,000 I.U. penicillin, 10,000 μg ml^−1^ streptomycin, 25 mg mL^−1^, Amphotericin B) was added to experimental vessels to discourage bacterial growth. After 24 h, larvae were fed 4 × 10^4^ cells mL^−1^ of *Isochrysis* spp. daily. Complete water changes were conducted bi-weekly, during which larvae were carefully passaged over a 64 μm mesh, rinsed, and re-suspended with 0.2 μm filtered-seawater. Temperature and pH_T_ were measured daily as described above. Experiments were completed once the majority of larvae metamorphosed in control treatments at which point larvae were preserved in a phosphate buffered formalin (3% v/v) solution. Survival, percent metamorphosis, and final length of larvae were determined using an inverted microscope coupled with a digital Nikon® camera and image analysis software.

### Multiple stressor experiments

Additional experiments were conducted with each cohort of larvae (Fig. 7) to assess how the exposure of adult bivalves to low pH during gametogenesis transgenerationally influenced the vulnerability of larvae to additional stressors including elevated temperature, food-limitation, and the harmful alga, *C. polykrikoides*. Upon fertilization, larvae were subjected to normal or undersaturated carbonate chemistry treatments for 48 h in separate 2-L experimental vessels (*n* = 3 replicates per treatment^−1^) as described above. After 48 h, subsets of larvae (*n* = 10 larvae replicate^−1^ treatment^−1^) from each experimental treatment were transferred to each well of a 10 mL, 6-well, microplate (6 wells treatment^−1^) for trials involving additional stressors. Clam and scallop larvae were exposed to 300 and 1000 cells mL^−1^ of *C. polykrikoides*, respectively (strain = CP1; ref. 74), cell densities representative of moderate blooms in an ecosystem setting^74^. For each trial, no-algae controls were included whereby larvae were exposed to algal growth medium (GSe) only. All plates were incubated at 24°C (6 wells treatment^−1^). Cultures used for trials were maintained at exponential growth phases in GSe medium and incubated on 12:12 light:dark cycle at a light intensity of ~100 μmol quanta^−1^ s^−1^ ^63^. An antibiotic solution (see above) was included to prevent bacterial contamination during experiments. Survival and activity of larvae were monitored daily using an inverted microscope.

For temperature and food-limitation exposures, larvae added to 6-well microplates, were gently placed into temperature-controlled water baths at 24 (control), 27, and 31°C. Larvae within ‘fed’ treatments (larval clams only) were provided *Isochrysis* spp. as a food source (detailed above), whereas larvae within food-limited treatments were starved. Survival and activity of larvae was monitored daily as described above.

### Statistical analysis

All statistical analyses were conducted using Rstudio© statistical software (www.rstudio.com). The effects of larval and adult carbonate chemistry, in addition to potential interactions between treatments, on the survival, length, and metamorphosis of shellfish larvae within acidification experiments were analyzed using a two-way analysis of variance (ANOVA). Post-hoc comparisons were performed and *p*-values adjusted accordingly for multiple comparisons using Tukey’s honest significant difference test (Tukey HSD). Assumptions of a normal distribution and homoscedasticity were confirmed using Shapiro-Wilk and Levene’s tests, respectively. When data failed to conform to a normal distribution with equal variance, an arcsin-square-root transformation was applied. Differences among egg sizes between low and ambient pH cohorts were assessed using a Welch’s t-test. Differences in survival among larval scallop cohorts exposed to elevated temperatures were assessed using a three-way ANOVA (main effects = adult and larval carbonate chemistry and temperature). Survival among the remaining larval trials (additional stressors) failed to conform to a normal distribution and/or display equal variance among replicates, despite transformation. Hence, a generalized linear model (GLM; three-way comparisons = adult and larval carbonate chemistry, and temperature or harmful algal exposure) was fit to the data using a binomial distribution (family = binomial). Multiple comparisons were assessed with Tukey’s procedure for multiple comparisons using the general linear hypothesis test function (function = glht) within the multiple comparisons package (package = multcomp; www.cran.r-project.org). All results were deemed significant at α ≤ 0.05.

### Data Availability

Data generated and analyzed during the current study are available from the corresponding author upon request.

## Electronic supplementary material

Supplementary Information

## References

[1] Doney SC, Schimel DS (2007). Carbon and Climate System Coupling on Timescales from the Precambrian to the Anthropocene*. Annual Review of Environment and Resources.

[2] IPCC. Climate change 2014: Synthesis report. Contribution of working groups I, II and III to the Fifth Assessment Report of the Intergovernmental Panel on Climate Change. (eds Pachauri, R. K. & Meyer, L. A.) (2014).

[3] Honisch B (2012). The geological record of ocean acidification. Science.

[4] Heogh-Guldberg, O. *et al*. The Ocean. In: *Climate Change 2014: Impacts, Adaptation, and Vulnerability. Part B: Regional Aspects. Contribution of Working Group II to the Fifth Assessment Report of the Intergovernmental Panel on Climate Change* (eds Barros, V.R., *et al*.). Cambridge University Press (2014).

[5] Solomon, S. *The physical science basis: Contribution of Working Group I to the fourth assessment report of the Intergovernmental Panel on Climate Change*. Cambridge University Press (2007).

[6] Doney SC (2012). Climate change impacts on marine ecosystems. Annual review of marine science.

[7] Parker LM (2012). Adult exposure influences offspring response to ocean acidification in oysters. Global change biology.

[8] Ross PM, Parker L, Byrne M (2016). Transgenerational responses of molluscs and echinoderms to changing ocean conditions. ICES Journal of Marine Science: Journal du Conseil.

[9] Salinas S, Munch SB (2012). Thermal legacies: transgenerational effects of temperature on growth in a vertebrate. Ecology letters.

[10] Byrne M. Impact of ocean warming and ocean acidification on marine invertebrate life history stages: vulnerabilities and potential for persistence in a changing ocean. In: *Oceanography and Marine Biollogy: An Annual Review* (eds Gibson, R., Atkinson, R., Gordon, J., Smith, I. & Hughes, D.). CRC Press (2011).

[11] Waldbusser GG, Salisbury JE (2014). Ocean acidification in the coastal zone from an organism’s perspective: multiple system parameters, frequency domains, and habitats. Annual review of marine science.

[12] Hamdoun A, Epel D (2007). Embryo stability and vulnerability in an always changing world. Proceedings of the National Academy of Sciences.

[13] Munday, P. L. Transgenerational acclimation of fishes to climate change and ocean acidification. *F1000prime reports* **6** (2014). 10.12703/P6-99 PMC4229724 25580253

[14] Mousseau T (1998). The adaptive significance of maternal effects. Trends in Ecology & Evolution.

[15] Marshall DJ, Keough MJ (2006). Complex life cycles and offspring provisioning in marine invertebrates. Integrative and Comparative Biology.

[16] Jablonka E, Raz G (2009). Transgenerational Epigenetic Inheritance: Prevalence, Mechanisms, and Implications for the Study of Heredity and Evolution. The Quarterly Review of Biology.

[17] Ho DH, Burggren WW (2010). Epigenetics and transgenerational transfer: a physiological perspective. Journal of Experimental Biology.

[18] Holeski LM, Jander G, Agrawal AA (2012). Transgenerational defense induction and epigenetic inheritance in plants. Trends in Ecology & Evolution.

[19] Sultan SE (2007). Development in context: the timely emergence of eco-devo. Trends in Ecology & Evolution.

[20] Schade FM, Clemmesen C, Mathias Wegner K (2014). Within- and transgenerational effects of ocean acidification on life history of marine three-spined stickleback (*Gasterosteus aculeatus*). Marine Biology.

[21] Marshall DJ (2008). Transgenerational Plasticity in the Sea: Context-Dependent Maternal Effects across the Life History. Ecology.

[22] Baumann H, Wallace RB, Tagliaferri T, Gobler CJ (2015). Large natural pH, CO2 and O2 fluctuations in a temperate tidal salt marsh on diel, seasonal, and interannual time scales. Estuaries and Coasts.

[23] Wallace RB, Baumann H, Grear JS, Aller RC, Gobler CJ (2014). Coastal ocean acidification: The other eutrophication problem. Estuarine, Coastal and Shelf Science.

[24] Cai W-J (2011). Acidification of subsurface coastal waters enhanced by eutrophication. Nat Geosci.

[25] Thomsen J (2017). Naturally acidified habitat selects for ocean acidification-tolerant mussels. Sci Adv.

[26] Harley CD (2006). The impacts of climate change in coastal marine systems. Ecology letters.

[27] Byrne M, Przeslawski R (2013). Multistressor impacts of warming and acidification of the ocean on marine invertebrates’ life histories. Integrative and Comparative Biology.

[28] Gobler CJ, DePasquale EL, Griffith AW, Baumann H (2014). Hypoxia and acidification have additive and synergistic negative effects on the growth, survival, and metamorphosis of early life stage bivalves. PLoS One.

[29] Melzner F (2013). Future ocean acidification will be amplified by hypoxia in coastal habitats. Marine Biology.

[30] Moore SK (2008). Impacts of climate variability and future climate change on harmful algal blooms and human health. Environmental health: a global access science source.

[31] Gobler, C. J. *et al*. Ocean warming since 1982 has expanded the niche of toxic algal blooms in the North Atlantic and North Pacific Oceans. *Proceedings of the National Academy of Sciences* In press (2017). 10.1073/pnas.1619575114 PMC5441705 28439007

[32] Behrenfeld MJ (2006). Climate-driven trends in contemporary ocean productivity. Nature.

[33] Doney SC (2006). Oceanography: Plankton in a warmer world. Nature.

[34] Talmage SC, Gobler CJ (2009). The effects of elevated carbon dioxide concentrations on the metamorphosis, size, and survival of larval hard clams (*Mercenaria mercenaria*), bay scallops (*Argopecten irradians*), and Eastern oysters (*Crassostrea virginica*). Limnology and Oceanography.

[35] Waldbusser GG (2015). Ocean acidification has multiple modes of action on bivalve larvae. PLoS One.

[36] Talmage SC, Gobler CJ (2011). Effects of elevated temperature and carbon dioxide on the growth and survival of larvae and juveniles of three species of northwest Atlantic bivalves. PLoS One.

[37] Waldbusser GG (2014). Saturation-state sensitivity of marine bivalve larvae to ocean acidification. Nature Climate Change.

[38] Gazeau F (2010). Effect of ocean acidification on the early life stages of the blue mussel *Mytilus edulis*. Biogeosciences.

[39] Fegley, S. R. Demography and dynamics of hard clam populations. In: *Biology fo the hard clam* (eds Kraeuter, J. N. & Castagna, M.). Elsevier Science (2001).

[40] Brand AR. Scallop Ecology: Distribution and Behaviour. In: *Scallops: Biology, Ecology, and Aquaculture* (eds Shumway, S. E. & Parsons, G. J.) Elsevier (2011).

[41] Wang ZA (2013). The marine inorganic carbon system along the Gulf of Mexico and Atlantic coasts of the United States: Insights from a transregional coastal carbon study. Limnology and Oceanography.

[42] Karmalkar AV, Bradley RS (2017). Consequences of Global Warming of 1.5°C and 2°C for Regional Temperature and Precipitation Changes in the Contiguous United States. PLoS One.

[43] Ekstrom JA (2015). Vulnerability and adaptation of US shellfisheries to ocean acidification. Nature Climate Change.

[44] Talmage SC, Gobler CJ (2010). Effects of past, present, and future ocean carbon dioxide concentrations on the growth and survival of larval shellfish. Proceedings of the National Academy of Sciences.

[45] Clark HR, Gobler CJ (2016). Diurnal fluctuations in CO2 and dissolved oxygen concentrations do not provide a refuge from hypoxia and acidification for early-life-stage bivalves. Marine Ecology Progress Series.

[46] Leverone JR, Shumway SE, Blake NJ (2007). Comparative effects of the toxic dinoflagellate *Karenia brevis* on clearance rates in juveniles of four bivalve molluscs from Florida, USA. Toxicon.

[47] Leverone JR, Blake NJ, Pierce RH, Shumway SE (2006). Effects of the dinoflagellate *Karenia brevis* on larval development in three species of bivalve mollusc from Florida. Toxicon.

[48] Fitzer SC, Cusack M, Phoenix VR, Kamenos NA (2014). Ocean acidification reduces the crystallographic control in juvenile mussel shells. Journal of structural biology.

[49] Eversole A. G. Reproduction in *Mercenaria mercenaria* In: *The Biology of the* Hard Clam (eds Kraeuter, J. N. & Castagna, M.). Elsevier (2001).

[50] Parker, L. M. *et al*. Adult exposure to ocean acidification is maladaptive for larvae of the Sydney rock oyster *Saccostrea glomerata* in the presence of multiple stressors. *Biology Letters* **13** (2017). 10.1098/rsbl.2016.0798 PMC5326511 28202683

[51] Allen RM, Buckley YM, Marshall DJ (2008). Offspring size plasticity in response to intraspecific competition: an adaptive maternal effect across life-history stages. The American naturalist.

[52] Podolsky RD (2004). Life-history consequences of investment in free-spawned eggs and their accessory coats. The American naturalist.

[53] Moran AL, McAlister JS (2009). Egg size as a life history character of marine invertebrates: Is it all it’s cracked up to be?. The Biological Bulletin.

[54] Pörtner HO, Farrell AP (2008). Physiology and climate change. Science.

[55] Gallager SM, Mann R, Sasaki GC (1986). Lipid as an index of growth and viability in three species of bivalve larvae. Aquaculture.

[56] Melzner F (2011). Food supply and seawater *p*CO2 impact calcification and internal shell dissolution in the blue mussel *Mytilus edulis*. PLoS One.

[57] Glibert PM, Dennett JC, Goldman JC (1985). Inorganic carbon uptake by phytoplankton in Vineyard Sound, Massachusetts. II. Comparative primary productivity and nutritional status of winter and summer assemblages. Jounral of Experimental Marine Biology and Ecology.

[58] Rose JM, Carron DA (2007). Does low temperature constrain the growth rates of heterotrophic protists? Evidence and implications for algal blooms in cold waters. Limnology and Oceanography.

[59] Roemmich D, McGowan J (1995). Climatic warming and the decline of zooplankton in the california current. Science.

[60] Yu BP (1994). Cellular defenses against damage from reactive oxygen species. Physiological reviews.

[61] Kim CS, Lee SG, Lee CK, Kim HG, Jung J (1999). Reactive oxygen species as causative agents in the ichthyotoxicity of the red tide dinoflagellate *Cochlodinium polykrikoides*. J Plankton Res.

[62] Kim CS, Lee SG, Kim HG (2000). Biochemical responses of fish exposed to a harmful dinoflagellate *Cochlodinium polykrikoides*. Journal of Experimental Marine Biology and Ecology.

[63] Tang YZ, Gobler CJ (2009). Characterization of the toxicity of *Cochlodinium polykrikoides* isolates from Northeast US estuaries to finfish and shellfish. Harmful Algae.

[64] Griffith AW, Gobler CJ (2016). Temperature Controls the toxicity of the icthyotoxic dinoflagellate. Cochlodinium polykrikoides. Marine Ecology Progress Series.

[65] Coen LD (2007). Ecosystem services related to oyster restoration. Marine Ecology Progress Series.

[66] Officer CB, Smayda TJ, Mann R (1982). Benthic Filter Feeding - a Natural Eutrophication Control. Marine Ecology Progress Series.

[67] Cerrato RM, Caron DA, Lonsdale DJ, Rose JM, Schaffner RA (2004). Effect of the northern quahog *Mercenaria mercenaria* on the development of blooms of the brown tide alga *Aureococcus anophagefferens*. Marine Ecology Progress Series.

[68] O’Leary, C., Nye, J., Tettelbach, S., Gobler, C., Grear, J. Effects of coastal acidification on North Atlantic bivalves: Scaling laboratory experiments to *in situ* populations. *Submitted* (2017). 10.3354/meps13140 PMC8193825 34121786

[69] Tettelbach ST, Smith CF, Smolowitz R, Tetrault K, Dumais S (1999). Evidence for fall spawning of nothern bay scallops *Argopecten irradians irradians* (Lamarck 1819) in New York. Journal of Shellfish Research.

[70] Kraeuter, J. N., Castagna, M. *Bilogy of the hard clam*. Elsevier (2001).

[71] Helm, M. M., Bourne, N. & Lovatelli, A. *Hatchery culture of bivalves: a practical manual*. Food and agriculture organization of the United Nations (2004).

[72] Bricelj VM, Epp J, Malouf RE (1987). Intraspecific variation in reproductive and somatic growth cycles of bay scallops *Argopecten-Irradians*. Marine Ecology Progress Series.

[73] Millero FJ (2010). Carbonate constants for estuarine waters. Marine and Freshwater Research.

[74] Gobler CJ (2008). Characterization, dynamics, and ecological impacts of harmful *Cochlodinium polykrikoides* blooms on eastern Long Island, NY, USA. Harmful Algae.

