# Supplementary material for: Transgenerational exposure of North Atlantic bivalves to ocean acidification renders offspring more vulnerable to low pH and additional stressors
Source: Sci Rep. 2017 Sep 12;7:11394. doi: 10.1038/s41598-017-11442-3 (PMC5595845; doi:10.1038/s41598-017-11442-3)
Supplement: Supplementary file 1 — Supplementary Information [file 41598_2017_11442_MOESM1_ESM.pdf]

Supplemental Information:

Transgenerational exposure of North Atlantic bivalves to ocean acidification renders offspring more vulnerable to low pH and additional stressors

Andrew W. Griffith[1] and Christopher J. Gobler[1*]

[1]School of Marine and Atmospheric Sciences, Stony Brook University, Southampton, NY, 11968

***Corresponding author**: Christopher J. Gobler; Phone: 1-631-632-5043; email: christopher.gobler@stonybrook.edu

Supplementary Tables:

Supplementary Table S1. Mean (± standard deviation) pH_T, pCO_2, aragonite and calcite saturation state ($\Omega_{aragonite}$, $\Omega_{calcite}$), total dissolved inorganic carbon (∑DIC), [CO_3^{2-}], and total alkalinity (TA) during clam and scallop conditioning periods.

Conditioning carbonate chemistry				
Parameter	Bay scallop (*Argopecten irradians*)		Hard clam (*Mercenaria mercenaria*)	
	Ambient CO_2	Elevated CO_2	Ambient CO_2	Elevated CO_2
pH_T	7.88 ± 0.07	7.42 ± 0.09	7.81 ± 0.05	7.35 ± 0.06
pCO_2 (µatm)	644.59 ± 7.45	2539.66 ± 189.42	567.8 ± 79.3	2651.2 ± 271.2
$\Omega_{aragonite}$	1.60 ± 0.02	0.55 ± 0.02	2.39 ± 0.433	0.66 ± 0.05
$\Omega_{calcite}$	2.50 ± 0.03	0.86 ± 0.04	3.69 ± 0.67	1.03 ± 0.08
∑DIC (µmol L^{-1})	2036.90 ± 24.85	2345.18 ± 31.41	1854.19 ± 160.4	2225.32 ± 122.2
CO_3^{2-} (µmol L^{-1})	101.62 ± 0.04	35.18 ± 1.42	148.10 ± 26.85	41.12 ± 3.06
TA (µmol L^{-1})	2165.40 ± 25.86	2307.53 ± 22.96	2013.4 ± 193.6	2194.53 ± 125.6

Supplementary Table S2. Mean (± standard deviation) $pH_{T,}$ pCO_2, aragonite and calcite saturation state ($\Omega_{aragonite,}$ $\Omega_{calcite}$), total dissolved inorganic carbon (∑DIC), [CO_3^{2-}], and total alkalinity (TA) during acidification trails with larval hard clams.

Hard clam carbonate chemistry (adult CO_2 conditions -> larval CO_2 conditions)				
Parameter	Ambient -> ambient	Ambient -> elevated	Elevated -> ambient	Elevated -> elevated
pH_{NBS}	8.13 ± 0.10	7.40 ± 0.09	8.11 ± 0.21	7.42 ± 0.10
pCO_2 (μatm)	512.5 ± 35	2161.70 ± 63	512.35 ± 35	2161.70 ± 63
$\Omega_{aragonite}$	1.9 ± 0	0.55 ± 0.07	1.9 ± 0.	0.55 ± 0.07
$\Omega_{calcite}$	2.9 ± 0	0.85 ± 0.07	2.9 ± 0	0.85 ± 0.07
∑DIC (μmol L^{-1})	1901 ± 79.19	2089.5 ± 74.24	1901 ± 79.19	2089.5 ± 74.24
CO_3^{2-} (μmol L^{-1})	120 ± 0.71	36 ± 2.7	120 ± 0.71	36 ± 2.7
TA (μmol L^{-1})	2065 ± 72.12	2072 ± 78.48	2065 ± 72.12	2072 ± 78.48

Supplementary Table S3. Mean (± standard deviation) pH_T, pCO_2, aragonite and calcite saturation state ($\Omega_{aragonite}$, $\Omega_{calcite}$), total dissolved inorganic carbon (∑DIC), [CO_3^{2-}], and total alkalinity (TA) during acidification trails with larval scallops.

Bay scallop carbonate chemistry (adult CO_2 conditions -> larval CO_2 conditions)				
Parameter	Ambient -> ambient	Ambient -> elevated	Elevated -> ambient	Elevated -> elevated
pH_T	7.90 ± 0.00	7.41 ± 0.02	7.90 ± 0.01	7.37 ± 0.02
pCO_2 (µatm)	525.14 ± 17.48	1859.21 ± 71.59	607.06 ± 16.08	2482.34 ± 164.84
$\Omega_{aragonite}$	2.14 ± 0.10	0.77 ± 0.04	2.43 ± 0.05	0.85 ± 0.07
$\Omega_{calcite}$	3.29 ± 0.16	1.18 ± 0.05	3.74 ± 0.07	1.31 ± 0.11
∑DIC (µmol L^{-1})	1802.12 ± 70.89	1979.18 ± 43.23	2065.55 ± 5.21	2399.82 ± 167.17
CO_3^{2-} (µmol L^{-1})	96.12 ± 4.61	34± 1.71	109.40 ± 2.22	37.52 ± 3.27
TA (µmol L^{-1})	1979.36 ± 77.02	1981.23 ± 50.14	2258.22 ± 3.77	2396.89 ± 167.30
